# Analysis of Pectin-derived Monosaccharides from *Arabidopsis* Using GC–MS

**DOI:** 10.21769/BioProtoc.4746

**Published:** 2023-08-20

**Authors:** Patricia Scholz, Kent D Chapman, Till Ischebeck, Athanas Guzha

**Affiliations:** 1 Department for Plant Biochemistry, Albrecht-von-Haller-Institute for Plant Sciences, University of Goettingen, Goettingen, Germany; 2 Department of Biological Sciences and BioDiscovery Institute, University of North Texas, Denton, TX, USA; 3 Institute of Plant Biology and Biotechnology (IBBP), Green Biotechnology, University of Münster, Münster, Germany

**Keywords:** Cell wall, Pectin, Gas chromatography–mass spectrometry, Monosaccharide, *Arabidopsis*

## Abstract

Pectin is a complex polysaccharide present in the plant cell wall, whose composition is constantly remodelled to adapt to environmental or developmental changes. Mutants with altered pectin composition have been reported to exhibit altered stress or pathogen resistance. Understanding the link between mutant phenotypes and their pectin composition requires robust analytical methods to detect changes in the relative monosaccharide composition. Here, we describe a quick and efficient gas chromatography–mass spectrometry (GC–MS)-based method that allows the differential analysis of pectin monosaccharide composition in plants under different conditions or between mutant plants and their respective wild types. Pectin is extracted from seed mucilage or from the alcohol-insoluble residue prepared from leaves or other organs and is subsequently hydrolysed with trifluoracetic acid. The resulting acidic and neutral monosaccharides are then derivatised and measured simultaneously by GC–MS.

Key features

Comparative analysis of monosaccharide content in *Arabidopsis*-derived pectin between different genotypes or different treatments.

Procedures for two sources of pectin are shown: seed coat mucilage and alcohol-insoluble residue.

Allows quick analyses of neutral and acidic monosaccharides simultaneously.

Graphical overview

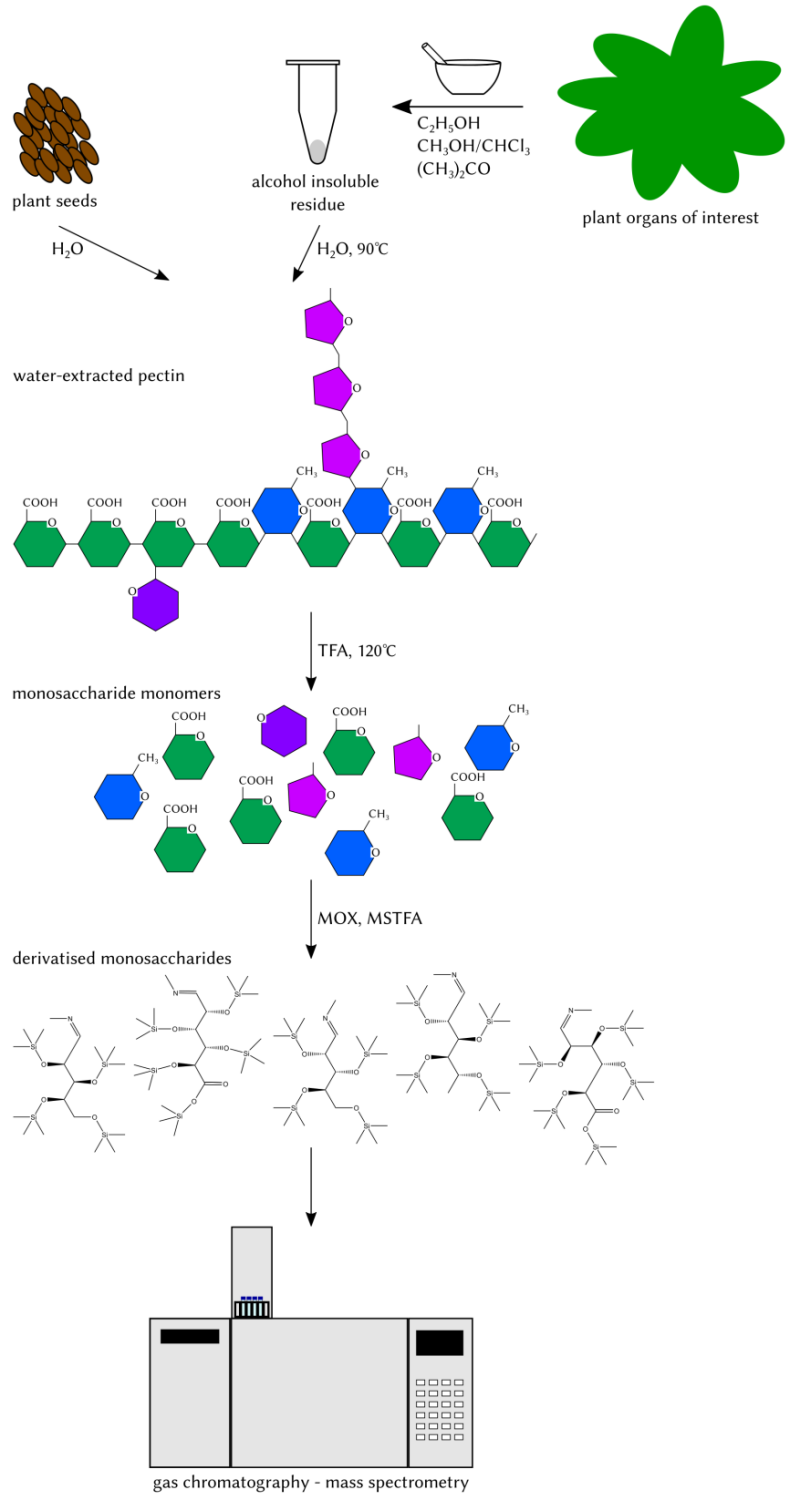

## Background

Primary cell walls consist of up to 90% polysaccharides including cellulose, hemicelluloses, and pectin (Pettolino et al., 2012; Höfte and Voxeur, 2017). Of those, pectin is characterised by high amounts of D-galacturonic acid, which forms the backbone in the pectic polysaccharides homogalacturonan and rhamnogalacturonan II. In a third pectic polysaccharide, rhamnogalacturonan I, the backbone consists of disaccharide units of D-galacturonic acid linked to L-rhamnose (Atmodjo et al., 2013). Further mono- or oligosaccharide side chains are linked to the backbone, resulting in a chemically complex structure (Harholt et al., 2010; Atmodjo et al., 2013).

Pectin is the most abundant component of primary cell walls of many plant species and performs diverse functional roles, including the response to infection by various pathogens (Shin et al., 2021). In this context, it was reported that an *Arabidopsis* double mutant line, disrupted in the two galacturonic acid–producing enzymes GLUCURONATE 4-EPIMERASE 1 and 6, contained less homogalacturonan and concomitantly showed higher susceptibility towards infection with the pathogens *Pseudomonas syringae* pv *maculicola* ES4326 and *Botrytis cinerea* isolates (Bethke et al., 2016). Also, modifications of the pectin backbone have been connected to plant–pathogen interactions. In *Arabidopsis*, POWDERY MILDEW RESISTANT5 (*PMR5*) is responsible for the acetylation of galacturonic acid, and the *pmr5* mutant has been shown to possess enhanced resistance to powdery mildew (Vogel et al., 2004). Furthermore, the *bxl4 Arabidopsis* mutant that lost enzyme activity of BETA-XYLOSIDOSE4 (BXL4) exhibited increased susceptibility to the fungal pathogen *B. cinerea*. BXL4 has been shown to possess activities on cell wall–derived arabinans and xyloses (Guzha et al., 2022).

Given the reported effects of changes in pectin abundance and composition, fast and straightforward methods to compare the relative composition of pectin in plants under different conditions or of different genotypes are essential. Different methods for compositional analysis have been described so far, including colorimetric assays, gas chromatography–mass spectrometry (GC–MS), and high-pressure liquid chromatography-based approaches (Pettolino et al., 2012; Biswal et al., 2017; Bethke and Glazebrook, 2019; Dean et al., 2019). Among those, GC–MS-based methods require analytes volatile enough for GC separation. Two main methods to obtain volatile analytes are described in the literature: reduction and acetylation to alditol acetates or acidic methanolysis of the sample followed by trimethylsilylation. The latter procedure offers the advantage of analysing neutral and acidic monosaccharides simultaneously; however, methanolysis is a time-consuming process with incubation times of 18 h (Biswal et al., 2017). We therefore intended to combine shorter sample processing times with the simultaneous study of neutral and acidic monosaccharides.

We describe a GC–MS-based analysis of cell wall pectin extracted from cell wall samples as alcohol-insoluble residue (AIR) or seed coat mucilage by water extraction. Subsequently, extracted pectin is hydrolysed with trifluoracetic acid (TFA) that can be evaporated to leave the monosaccharide monomers. These are then derivatised with *O*-methylhydroxylamine hydrochloride (MOX) and *N*-methyl-*N*-(trimethylsilyl)trifluoroacetamide (MSTFA) to yield volatile analytes to be analysed by GC–MS.

## Materials and reagents

**Reagents**

1. Ultrapure water (prepared, for example, with a water purification system)
2. Liquid nitrogen
3. Nitrogen (N_2_) gas
4. Acetone (Carl Roth, catalog number: 9372.5)
5. Chloroform (Carl Roth, catalog number: 7331.1)
6. Undenatured ethanol
7. Methanol (Fisher Scientific, catalog number: 10124490)
8. Pyridine anhydrous (Sigma-Aldrich, catalog number: 270970)
9. Trifluoroacetic acid (TFA) (Sigma-Aldrich, catalog number: 302031)
10. *O*-Methylhydroxylamine hydrochloride (MOX) (Sigma-Aldrich, catalog number: 226904)
11. *N*-Methyl-*N*-trimethylsilyltrifluoroacetamide (MSTFA) (Sigma-Aldrich, catalog number: 69479)
12. *allo*-Inositol (Sigma-Aldrich, catalog number: 468088)
13. L-Arabinose (Sigma-Aldrich, catalog number: A91906)
14. L-Fucose (Sigma-Aldrich, catalog number: F2252)
15. D-Galactose (Carl Roth, catalog number: 4987.2)
16. D-Galacturonic acid monohydrate (Sigma-Aldrich, catalog number: 48280)
17. D-Glucose (Carl Roth, catalog number: 6780.2)
18. D-Glucuronic acid sodium salt monohydrate (Sigma-Aldrich, catalog number: G8645)
19. D-Mannose (Sigma-Aldrich, catalog number: M8574)
20. L-Rhamnose monohydrate (Carl Roth, catalog number: 4655.2)
21. D-Xylose (Sigma-Aldrich, catalog number: X3877)

**Solutions**

1. 70% ethanol (see Recipes)
2. 2 M trifluoroacetic acid (TFA) (see Recipes)
3. *allo*-Inositol (see Recipes)
4. Monosaccharide standards (see Recipes)
5. *O*-Methylhydroxylamine hydrochloride (MOX) derivatisation solution (see Recipes)

## Recipes

1. **70% ethanol**
  **Table:** 

Reagent	Final concentration	Quantity
Ethanol (absolute)	70%	70 mL
H_2_O	n/a	30 mL
Total	n/a	100 mL
2. **2 M trifluoracetic acid (TFA)**
  **Table:** 

Reagent	Final concentration	Quantity
TFA (13 M)	2 M	1.54 mL
H_2_O	n/a	8.46 mL
Total	n/a	10 mL
  Add the acid slowly to ultrapure water. 10 mL of 2 M TFA solution is enough for the hydrolysis of 30 samples. **Caution:** TFA is corrosive and attacks skin, eyes, and mucous membranes. Wear chemical-resistant gloves and safety goggles and work with TFA only under a fume hood.
3. ***allo*-Inositol**
  **Table:** 

Reagent	Final concentration	Quantity
*allo*-Inositol	50 μg/mL	5 mg
H_2_O	n/a	100 mL
Total	n/a	100 mL
4. **Monosaccharide standards**
  **Table:** 

Reagent	Final concentration	Quantity
L-Arabinose	250 mM	37.53 mg in 1 mL of water
D-Xylose	250 mM	37.53 mg in 1 mL of water
L-Fucose	250 mM	41.04 mg in 1 mL of water
L-Rhamnose	250 mM	45.54 mg in 1 mL of water
D-Galactose	250 mM	45.04 mg in 1 mL of water
D-Glucose	250 mM	45.04 mg in 1 mL of water
D-Mannose	250 mM	45.04 mg in 1 mL of water
D-Galacturonic acid	250 mM	53.04 mg in 1 mL of water
D-Glucuronic acid	250 mM	58.54 mg in 1 mL of water
  Note down the actual mass that you weighed in and adjust the volume using the following formula:
$$V\;\left(water\right)=1\;mL\times \frac{actual\;mass\;in\;mg}{theoretical\;mass\;in\;mg\;as\;given\;in\;Table\;1}$$
5. ***O*-Methylhydroxylamine hydrochloride (MOX) derivatisation solution (see General note 3)**
  **Table:** 

Reagent	Final concentration	Quantity
MOX	30 mg/mL	15 mg
Pyridine anhydrous	n/a	0.5 mL
Total	n/a	0.5 mL
  **Caution:** Pyridine is harmful if swallowed, in contact with skin, or if inhaled. Wear chemical-resistant gloves and work with pyridine and MOX solutions only under a fume hood. Use solvent-resistant tips for pipetting.

**Laboratory supplies**

1. 10 cm plant pots
2. Soil: Einheitserde SPECIAL Vermehrung, Patzer Erden, Sinntal-Altengronau, Germany; medium clay content, contains peat, perlite, 1% nutrient salts, trace elements and iron, pH 5.8
3. Chemical-resistant gloves
4. Safety goggles
5. Solvent-resistant pipette tips [Biozym, catalog numbers: 693010 (10 μL), 692069 (200 μL), 692078 (1,000 μL)]
6. Vials, inserts, and lids for GC–MS [Macherey-Nagel, catalog numbers: 702282 (vials), 702716 (inserts), 702287.1 (lids)]
7. Glass beads, 5 mm diameter (Carl Roth, catalog number: HH56.1)
8. 2 mL microcentrifuge tube (Sarstedt, catalog number: 72.695.500)
9. 1.5 mL microcentrifuge tube (Sarstedt, catalog number: 72.690.001)
10. DURAN^®^ culture tubes with screw cap (VWR, catalog number: 391-4022)

## Equipment

1. Growth chamber with light and temperature controls
2. Oven for sterilization of soil at 80 °C
3. Fume hood
4. Gas chromatography–mass spectrometry (GC–MS) system:
  Agilent Technologies 7890B GC-System coupled to 5977B MSD quadrupole
  PAL3 Auto sampler system with Robotic Tool Change (RTC)
  HP-5ms Ultra Inert column (Agilent, catalog number: 19091S-433UI)
5. Nitrogen evaporator/sample concentrator
6. Analytical balance (Kern, model: 770-15)
7. Rotary shaker
8. Microcentrifuge
9. Heating block with shaker for 2 mL microcentrifuge tubes (HLC HTM 130, Haep Labor Consult)
10. Heating block for DURAN glass tubes (Liebisch^TM^ Monoblock)
11. Ball mill (Retsch MM400)
12. Optional: Mortar and pestle
13. Vortex mixer (Vortex-Genie 2)
14. Micropipettes
15. Acid resistant micropipette
16. Laboratory spatulas

## Software

1. GC/MSD MassHunter with MSD ChemStation Data Analysis (G1701FA F.01.03.2357; Agilent Technologies)
2. Microsoft Excel 2019 (Microsoft)

## Procedure

- **Plant growth**
  For individual experimental setup, we recommend plants to be grown according to your usual procedure. Pots with the various genotypes (3–4 pots, each with five plants per genotype) should be randomised, placed in the same tray, and grown together under the same conditions. As an example, the procedure to enable comparative pectin analysis on alcohol-insoluble residue (AIR) derived from leaves, as used in Guzha et al. (2022), is shown below.
  1. Prepare semi-sterile soil by heating it in the oven for 8 h at 80 °C.
  2. Transfer soil to 10 cm pots, water, and place 6–10 *Arabidopsis* seeds in the four corners and the centre of the pots. Cover with plastic dome.
  3. After stratification at 4 °C for two days, transfer to growth cabinet with short-day conditions (8 h light and 16 h darkness) at 22 °C, a relative humidity of 65%, and a light intensity of 120–140 μmol m^-2^·s^-1^. Remove the plastic dome after 3–4 days.
  4. Grow plants for 7–10 days, then thin out excess seedlings to leave five plants per pot (one plant per position).
  5. Grow until plants reach the desired growth stage depending on your experimental setup, e.g., 6-week-old plants for analysis of leaf AIR.
  A detailed description on plant growth and seed harvest for mucilage analysis is given in Dean et al. (2019) (see General note 1).
- **Preparation of alcohol insoluble residue (AIR)**
  The method used in this part of the protocol is adapted from Bethke et al. (2016) and involves the following steps:
  1. Flash-freeze the organs of interest (leaves, stems, roots, whole rosettes) in liquid nitrogen. Soil debris attached to roots or stems can be gently washed off using sterile distilled Milli-Q water.
*Note: To minimise starch contamination, leaf samples can be harvested after keeping the plants in the dark for 48 h, if the experimental setup allows (see General note 2).*
  2. Grind the frozen plant material using mortar and pestle or a ball mill. Keep the plant material frozen throughout grinding and make sure that the plant material is ground as fine as possible to minimize non–cell wall contamination. **Pause point:** The ground plant material can be stored at -80 °C.
  3. Transfer up to 100 mg of ground plant material in a pre-cooled 2 mL microcentrifuge tube. Keep samples cooled before the addition of ethanol in the next step.
  4. Add 1.5 mL of 70% (v/v) ethanol and vortex thoroughly.
  5. Centrifuge at 18,000× *g* for 10 min at room temperature and remove supernatant.
  6. Repeat steps B4 and B5 as described above.
  7. Add 1 mL of the solvent mixture chloroform/methanol 1:1 (v/v) and vortex thoroughly. **Caution:** Work with chloroform-containing solutions under a fume hood.
  8. Centrifuge at 18,000× *g* for 10 min at room temperature and remove supernatant. **Caution:** Removal of the chloroform-containing solution should be carried out under a fume hood.
  9. Repeat steps B7 and B8 two times as described above. After the final washing step, the plant material should have a grey to off-white colour, as all chlorophyll-containing fractions have been removed. Carry out additional washes with chloroform/methanol 1:1 (v/v) if the plant material is still green.
  10. Add 1 mL of acetone to the plant material and vortex thoroughly.
  11. Centrifuge at 18,000× *g* for 10 min at room temperature and remove supernatant.
  12. Air dry the resulting AIR at room temperature overnight. Keep samples in a dust-free environment to avoid contamination while drying.
  13. To avoid starch contamination, it is possible to carry out an α-amylase digest (see General note 2).
- **Extraction and hydrolysis of pectin**
  1. Pectin analysis from AIR
    - Prepare at least four replicates for each genotype or growth condition. Weigh 2 mg of AIR for each replicate and place in a 2 mL microcentrifuge tube. Record the exact weight of AIR placed in the microcentrifuge tube. Use AIR from plants grown at the same time and under the same conditions.
    - Add 1.4 mL of water to the AIR.
    - Add two glass beads (5 mm diameter) and homogenise AIR further with a ball mill for 1.5 min at 30 Hz.
    - Incubate samples for 2 h at 90 °C under constant vigorous shaking (speed #4 on HLC HTM 130).
    - Remove samples from heating block and allow the debris to settle for 5 min. Do not centrifuge the samples.
    - Transfer 1 mL of the supernatant to a DURAN^®^ glass culture tube with screw cap.
    - Dry the samples under a stream of N_2_ gas at 50 °C.
*Note: The drying step might take a while due to the aqueous nature of the sample.*
    - Add 300 μL of 2 M TFA to the dried samples. **Caution:** TFA is corrosive and attacks skin, eyes, and mucous membranes. Wear chemical-resistant gloves and safety goggles and work with TFA only under a fume hood.
    - Hydrolyse the samples for 1 h at 121 °C in a heating block for DURAN glass tubes.
    - After hydrolysis, evaporate the TFA under N_2_ stream.
    - Add 500 μL of water and 100 μL of the internal standard 0.05 mg/mL *allo*-inositol to the dried hydrolysis products. **Pause point:** For long-term storage, dissolved hydrolysis products can be kept at -20 °C for several months.
    - Sample preparation for subsequent measurements: dry 20 μL of the dissolved hydrolysis products under a stream of N_2_ gas.
    - Derivatise the dried samples with 15 μL of MOX (30 mg/mL in anhydrous pyridine) at room temperature overnight. Use freshly prepared MOX (see General note 3). Wear chemical-resistant gloves and work with pyridine and MOX solution only under a fume hood. Use solvent-resistant tips for pipetting.
    - After overnight derivatisation, add 30 μL of MSTFA and measure by GC–MS after at least 1 h incubation time at room temperature. Incubation time with MSTFA before GC–MS analysis should not exceed 6 h.
  1. Pectin analysis from mucilage
    - Prepare at least four replicates of each genotype. Weigh 5 mg of seeds for each replicate and transfer them to a 2 mL microcentrifuge tube. Record the exact mass of prepared seeds.
    - Add 1.4 mL of water to the seeds and vortex for a few seconds.
    - Place samples on a rotary shaker for 2 h to extract mucilage.
    - Remove the samples from the shaker and allow the seeds to settle for 1–2 min. Do not centrifuge the samples.
    - Transfer 1 mL of the supernatant to a DURAN glass tube.
    - Dry the samples under a stream of N_2_ gas at 50 °C.
*Note: The drying step might take a while due to the aqueous nature of the sample.*
    - Add 300 μL of 2 M TFA to the dried samples.
      **Caution:** TFA is corrosive and attacks skin, eyes, and mucous membranes. Wear chemical-resistant gloves and safety goggles and work with TFA only under a fume hood.
    - Hydrolyse the samples for 1 h at 121 °C in a heating block for DURAN glass tubes.
    - After hydrolysis, evaporate the TFA under N_2_ stream.
    - Add 500 μL of water and 100 μL of the internal standard 0.05 mg/mL *allo*-inositol to the dried hydrolysis products.
      **Pause point:** For long-term storage, dissolved hydrolysis products can be kept at -20 °C for several months.
    - Sample preparation for subsequent measurements: dry 20 μL of the dissolved hydrolysis products under a stream of N_2_ gas.
    - Derivatise the dried samples with 15 μL of MOX (30 mg/mL in anhydrous pyridine) at room temperature overnight. Use freshly prepared MOX (see Notes). Wear chemical-resistant gloves and work with pyridine and MOX solution only under a fume hood. Use solvent-resistant tips for pipetting.
    - After overnight derivatisation, add 30 μL of MSTFA and measure by GC–MS after at least 1 h incubation time at room temperature. Incubation time with MSTFA before GC–MS analysis should not exceed 6 h.
- **GC–MS analysis**
  1. Preparation of standards for calibration
    - From the stock solutions of the monosaccharides, prepare standard combinations of defined molar amounts by combining aliquots of dilutions from the respective stock solutions with the correct volume. (For example, for a mix of 1 nmol arabinose and 1 nmol galactose, combine 4 μL each of 1/1,000 dilutions of the stock solutions of arabinose and galactose.) Add 5 μL of 0.05 mg/mL *allo*-inositol.
      For less abundant pectic monosaccharides, prepare 3–5 different molar amount samples in the range of 1–5 nmol. For highly abundant pectic monosaccharides, prepare 3–5 different molar amount samples in the range of 20–100 nmol. Low or high abundance of the various monosaccharides depends on the sample; galacturonic acid will generally be highly abundant, whereas rhamnose is expected to be highly abundant in seed mucilage but less abundant in AIR from leaves. If the sample composition is unclear, it might be helpful to compare initial GC–MS results of a low and a high concentration of each standard with the sample result and then adjust the concentrations accordingly. You can combine different monosaccharide standards in the following combinations:
      1. Xylose, fucose, mannose
      2. Arabinose, galactose
      3. Glucose, glucuronic acid
      4. Rhamnose, galacturonic acid
      Prepare all standard combinations and different molar amounts in triplicates.
    - Dry the prepared standard solutions under a stream of N_2_ gas.
    - Derivatise the dried standards with 15 μL of MOX (30 mg/mL in anhydrous pyridine) at room temperature overnight. Use freshly prepared MOX solution (see General note 3). Wear chemical-resistant gloves and work with pyridine and MOX solution only under a fume hood. Use solvent-resistant tips for pipetting.
    - After overnight derivatisation, add 30 μL of MSTFA and analyse on GC–MS after at least 1 h incubation time. Incubation time with MSTFA before GC–MS analysis should not exceed 6 h.
  2. Monosaccharide analysis with GC-MS
    - Inject 1 μL of the derivatised sample with a split value of 10. Helium is used as carrier gas at a flow rate of 1 mL/min.
    - Use the following temperature gradient: 150 °C for 2 min, ramp to 250 °C at 5 K/min (20 min), ramp to 320 °C at 15 K/min (4.67 min), 320 °C for 3 min. The inlet temperature is set to 250 °C.
    - The transfer line temperature is set at 280 °C; ionisation is done with an electron energy of 70 eV and an ion source temperature of 230 °C. Mass spectra are recorded in an *m/z* range of 40–500.
  3. Data processing and peak integration
    Perform peak assignment and integration according to your usual procedure. Here, we describe exemplarily data processing and peak integration with MSD ChemStation Data Analysis.
    - Define compound list for the individual monosaccharides in the software MSD ChemStation Data Analysis. Use the GC–MS runs of the standards to determine retention times and characteristic fragment ions of the individual monosaccharides. Include the internal standard *allo*-inositol as additional compound.
*Note: For most monosaccharides, two peaks will be detected (Figure 1), as derivatisation with MOX yields two stereoisomers (compare retention indices in Table 1). For quantification, select the analyte peak without interference from other monosaccharide-derived analyte peaks. If there is no interference for any analyte peak of an individual monosaccharide, select the largest one.*
      **Figure 1.**
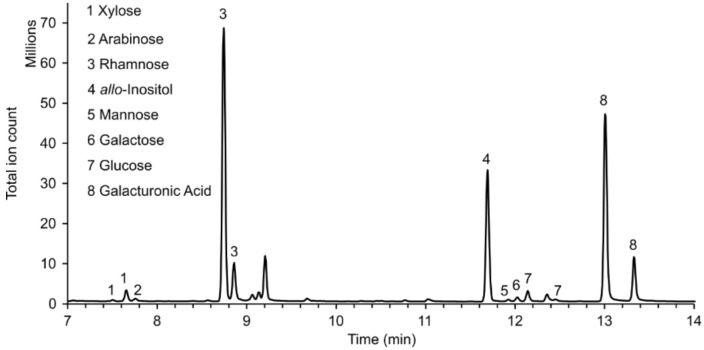
Example of the GC–MS chromatogram of monosaccharides from mucilage pectin as observed with MSD ChemStation Data Analysis. The vertical axis shows detected signals, while the horizontal axis shows the retention time in minutes after injection. *allo*-Inositol was used as internal standard (4). Fucose and glucuronic acid were not detected in this sample; the respective analyte peaks would have retention times of ca. 8.9 and 9.1 min for fucose, and 12.8 and 13.6 min for glucuronic acid. The peak at 9.1 min does not have the correct mass spectrum when compared with an external fucose standard.
      **Table 1.** GC–MS detection of individual monosaccharides. Possible target ions for quantification and the device-independent retention index are given in the middle columns. As an example, the retention times of the different monosaccharides as shown in Figure 1 are also given. Monosaccharide peaks that could not be detected in the respective sample are denoted as n.d.

	*m/z* quantitation ion	Retention index	Retention time in Figure 1
*allo*-Inositol	318	1908	11.7 min
L-Arabinose	307	1677	7.8 min
D-Xylose	307	1662, 1671	7.5 min, 7.7 min
L-Fucose	277	1744, 1756	both peaks n.d.
L-Rhamnose	277	1735, 1742	8.7 min, 8.9 min
D-Galactose	319	1928, 1950	12.0 min, second peak n.d.
D-Glucose	319	1935, 1954	12.1 min, second peak n.d.
D-Mannose	319	1922, 1937	11.9 min, second peak n.d.
D-Galacturonic acid	333	1986, 2004	13.0 min, 13.3 min
D-Glucuronic acid	333	1977, 1992	both peaks n.d.
    - Define one characteristic target ion as quantitation signal for all compounds. See Table 1 for possible quantitation ions of the derivatised analytes of the indicated monosaccharides.
    - The software will integrate the peaks of the target ions belonging to the different monosaccharides in the user-defined compound list. After automatic integration, it is important to check that monosaccharide peaks are correctly assigned, and peak areas are accurately integrated. If necessary, correct peak assignment and integration.
    - Re-integrate the peak intensities after correction of peak assignments or integration. Export the values for further analysis in Excel.

## Data analysis

The exported peak integration values can be further processed with Microsoft Excel or equivalent software. An example calculation to obtain the molar amount of xylose depicted in the sample of Figure 1 is displayed at the end of this section.

- **Analysis of standards**
  1. Paste the values obtained with MSD ChemStation Data Analysis into an Excel spreadsheet.
  2. Normalise each peak area of the different monosaccharide peaks to the internal standard *allo*-inositol. To that end, calculate the ratio of the respective peak areas and the integrated peak area of *allo*-inositol.
  3. Plot the normalised peak area vs. the molar amounts of the standard and perform a linear regression analysis on the plot. The R^2^ value should be close to 1.
  4. Use the LINEST function in Excel to calculate the parameters of a linear correlation between peak area and molar amount of the individual monosaccharide standards with the *least squares* method. The function will calculate the values *m* and *b* of the following model:
normalised peak area=m×molar amount+b
  5. Note down the respective values of *m* and *b* for each individual monosaccharide peak. These are required for sample analysis.
- **Sample analysis**
  1. As for the standards, paste the values obtained with MSD ChemStation Data Analysis into an Excel spreadsheet.
  2. Normalise the peak areas of the different monosaccharides to the internal standard *allo*-inositol.
    - Calculate the initial normalisation by forming the ratio between the peak area of the individual monosaccharides and the peak area of the standard *allo*-inositol.
    - In the standards, a total of 0.25 μg of *allo*-inositol was added (5 μL × 0.05 mg/mL), whereas in the samples 5 μg of *allo*-inositol (100 μL × 0.05 mg/mL) were added to the hydrolysis product. To correct against these different amounts of the standard, multiply the values for the normalised peak area obtained above (step B2a) with a correction factor of 20 (5 μg/0.25 μg = 20).
  3. Using the normalised peak area of the monosaccharides and the linear regression parameters *m* and *b* obtained above, you can now calculate the molar amounts of the individual monosaccharides with the following formula:
$$molar\;amount=\frac{normalised\;peak\;area-b}{m}$$
  4. Normalise the obtained molar amount to the initial sample mass of AIR or seeds. You can use the normalised molar amounts to determine the relative composition of your pectin samples. Calculate average and standard deviations for the percentages of the different monosaccharide compounds. You can also normalise the contribution of the less abundant monosaccharides to one of the dominating monosaccharides (see for example Guzha et al., 2022).
  The measured values, calculations, and calculated values for the second xylose peak of Figure 1 are shown below as an example (Table 2).

**Table 2.** Exemplary values for the calculation of molar amounts of xylose. The first column highlights the concentrations used for the external standard of xylose, all prepared in triplicates. The second and third columns show the integrated peak values as obtained from MSD ChemStation Data Analysis for xylose and *allo*-inositol, respectively. For the normalised values (column four), the ratio of values for xylose to the values for *allo*-inositol was calculated. In the lower section of the table, the averaged values for each concentration of the xylose standard are shown (Data analysis steps A1 and A2).

External standard	Peak area of xylose [AU]	Peak area of *allo*-inositol [AU]	Normalised values
1 nmol	20176173	89977965	0.22423
1 nmol	16685600	78745304	0.21189
1 nmol	15301979	76681503	0.19955
2 nmol	33336682	76622222	0.43507
2 nmol	34115172	73181461	0.46617
2 nmol	31752336	68480181	0.46367
3 nmol	46560441	60818809	0.76555
3 nmol	47484312	61529497	0.77173
3 nmol	52695435	70783534	0.74445
**Average normalised values**			
1 mol	0.21189		
2 mol	0.45497		
3 mol	0.76058		

With the LINEST function of Excel, the correlation between the concentration of the standards and the average normalised values was calculated, leading to the following parameters (Data analysis step A4):

m = 0.27434; b = -0.07287.

For the sample shown in Figure 1, the following peak areas were integrated:

Xylose peak 2: 4484042; *allo*-inositol: 100751631

This enabled the calculations shown below:

Normalised value according to B2a:

$$\frac{4484042}{100751631}=0.044506$$

Corrected normalised value according to B2b:

0.044506×20=0.89012

Molar amount of xylose according to B3:

$$\frac{0.89012-(-0.07287)}{0.27434}=3.51\;nmol$$

## Validation of protocol

This protocol or parts of it have been used in the following research articles: Guzha et al. (2022). Cell wall–localized BETA-XYLOSIDASE4 contributes to immunity of *Arabidopsis* against *Botrytis cinerea* (Figure 8E, 9F, S3A, S8, and S13).

## General notes and troubleshooting

1. For mucilage analysis, it is critical that the mother plants (wild type and mutants) are grown together at the same standard growth conditions. Water the plants until the final siliques turn yellow and begin to dry out. A detailed description of plant growth conditions for mucilage analysis can be found in Dean et al. (2019).
  Grow several independent seed sets (including wild type) and perform independent mucilage analyses to detect variations caused by slightly different growth conditions.
2. Starch contamination in the sample will cause excessive glucose signals. For leaf samples, plastidial starch accumulation can be avoided by keeping the plants in the dark for 48 h before harvest. Alternatively, starch can be removed by an α-amylase digest followed by an ethanol extraction after AIR preparation (Pettolino et al., 2012).
3. The derivatisation solution of 30 mg/mL MOX in anhydrous pyridine should not be older than three days to limit contaminations with water.
4. Calibration values obtained from one set of standards can be used for an extended time, as long as the sample type stays the same. In our hands, calibration values could be used for ~100 sample analyses.
